# LC-MS Identification of Rare Ethyl Esters in Botrytized Wines

**DOI:** 10.3390/molecules31203776

**Published:** 2026-10-21

**Authors:** Olga Stężycka, Daniel Górny, Olga Vyviurska, Ivan Špánik, Małgorzata Kasperkowiak, Magdalena Frańska

**Affiliations:** 1 Institute of Chemistry and Technical Electrochemistry, Poznań University of Technology, Berdychowo 4, 60-965 Poznań, Poland; olga.stezycka@doctorate.put.poznan.pl (O.S.);; 2 Faculty of Chemical and Food Technology, Institute of Analytical Chemistry, Slovak University of Technology in Bratislava, 81237 Bratislava, Slovakia; olga.vyviurska@stuba.sk (O.V.);; 3 Centre for Advanced Technologies, Adam Mickiewicz University, Uniwersytetu Poznańskiego 10, 61-614 Poznań, Poland

**Keywords:** botrytized wine, liquid chromatography, mass spectrometry, fragmentation pathways, relative peak intensity

## Abstract

The Tokaj region is one of the oldest and most famous European wine regions, located in two neighboring countries, Hungary and Slovakia, both of which are famous for their production of high-quality botrytized wines. Unfortunately, no analysis aimed at the identification of the most abundant monoethyl esters present in this type of wine has yet been made. In this paper, we present the LC-MS identification of the most abundant monoethyl esters of the common dicarboxylic aliphatic and phenolic acids in the botrytized Slovak and Hungarian wines, as well as the Slovak varietal wines, produced in the Tokaj wine region. French botrytized wines, produced in the Sauternes region, were also included in this study. Among the identified compounds, the unique esters, namely conjugates of tartaric acids and derivatives of cinnamic acid, were identified. The identification was based on the detection of the respective parent and product ions. Since the reports on the identification of the analyzed ethyl esters in wines are rare, their fragmentation pathways, including those of the identified isomers, were discussed in detail. Similar relative amounts of some of the identified unique esters in botrytized wines offer the potential of using these compounds as authenticity markers for this type of wine.

## 1. Introduction

Organic acids naturally occurring in wines react with ethanol; present in large excess over the former, and the respective esters are thus formed [1,2,3]. Esterification reaction, as well as ester hydrolysis (reversed reaction), can be affected by various parameters, e.g., pH, temperature, and other ingredients of a given wine [4]. Therefore, in the process of wine aging, an equilibrium is established between the acids and the esters, which has a considerable impact on the wine quality, namely its aroma and flavor [1,5]. There are a number of reports on the GC-MS analysis of fatty acid ethyl esters [6,7,8,9,10,11,12]. However, the main organic acids occurring in wine are dicarboxylic acids, namely tartaric and malic acids. Succinic acid and citric acid (tricarboxylic acid) are present as well, but their concentrations are usually lower than those of tartaric and malic acids [13]. Studies devoted to the analysis of ethyl esters of tartaric, malic, succinic, and citric acids in wines are less numerous than those on analysis of fatty acid ethyl esters [3,14,15,16,17]. Gas chromatography–mass spectrometry (GC-MS) is an appropriate method to analyze the completely esterified acids (volatile compounds), and, in turn, the di- or triethyl esters of di- and tricarboxylic acids [16,17]. Another group of organic acids present in wines are conjugates (formally esters) of tartaric acids and derivatives of cinnamic acid, namely caftaric acid (conjugate of tartaric and caffeic acid), coutaric acid (conjugate of tartaric and coumaric acid), and fertaric acid (conjugate of tartaric and ferulic acid) [18,19,20,21,22,23]. These acids also undergo esterification with ethanol, although, to the best of our knowledge, the reports on the formed esters are extremely rare. These compounds have been identified in the Riesling wine [24,25] and in white wine [26].

Botrytized wines, which belong to a special high-quality category of dessert wines, have a pleasant honey-like taste along with a distinctive fruit flavor. The use of berries infected with *Botrytis cinerea* in the production of wines significantly alters the organoleptic and chemical profile of the final product. There are a few regions in Europe where this type of wine is produced, such as the Tokaj wine region in Hungary and Slovakia, the Sauternes wine region in France, and the Burgenland wine region in Austria.

The objective of this work was the identification of the main monoethyl esters of dicarboxylic acids in botrytized and varietal wines. We performed liquid chromatography–mass spectrometry (LC-MS) analysis of botrytized wine samples produced in the Tokaj wine region (**SK_A–E**, **HU_A–D**) and in the Sauternes region (**FR_A–C**), as well as varietal wine samples (**SK_F–K**) produced in the Tokaj wine region (Table 1 and Table 2). In each sample, besides the expected organic acids, the respective monoethyl esters were identified, and are discussed in detail. On the basis of the mass spectrometric data obtained, i.e., fragmentation pathways of [M − H]^−^ and/or [M + H]^+^ ions, very rare compounds were identified.

## 2. Results and Discussion

### 2.1. LC-MS Identification of Organic Acids

**Scheme 1:**
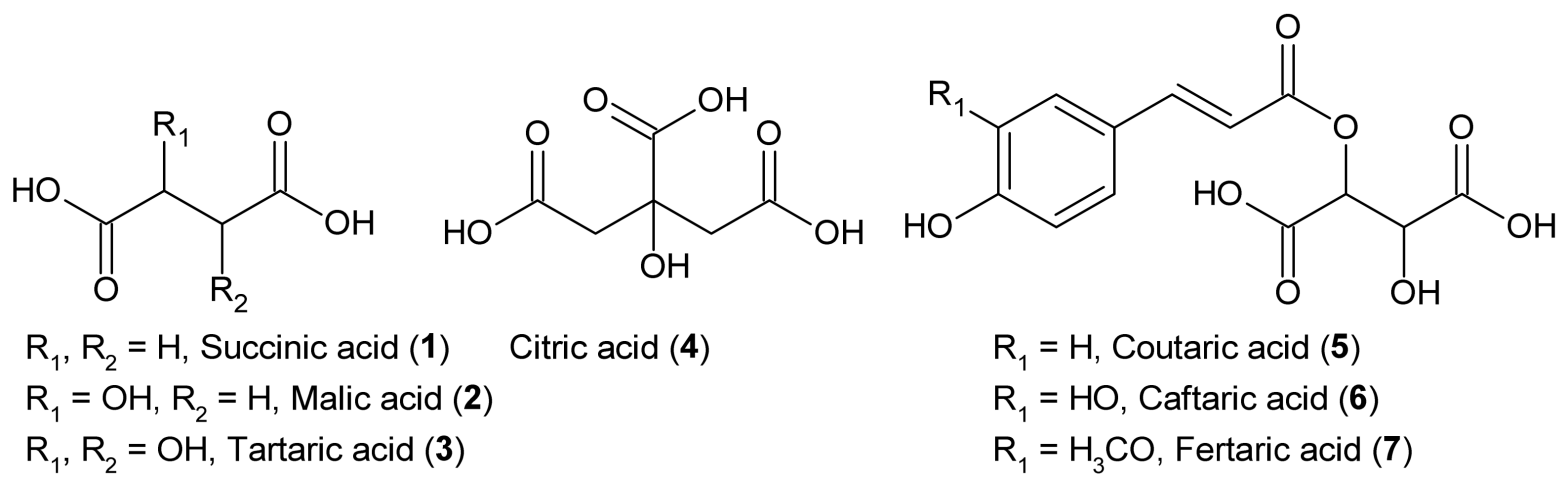
Structures of the detected dicarboxylic organic acids (**1**–**7**).

Although the aim of this work was to identify the monoethyl esters of the dicarboxylic acids, both aliphatic and phenolic acids (Scheme 1), whose presence was expected, were also detected.

Exemplary single-ion chromatograms of [M − H]^−^ ions of the identified acids obtained upon LC-MS analysis of the wine samples are shown in the Supplementary Material (Figures S1 and S2). Since acids **2**–**4** are hydrophilic compounds, under reversed-phase liquid chromatography, they are eluted quickly due to the weak interaction with the stationary phase; we deal with the resultant tailing peaks (in contrast to the peaks assigned to the other compounds studied, e.g., aromatic acids **5**–**7**). Furthermore, for citric acid (**4**), which is tricarboxylic and can exist in a number of dissociated/undissociated forms, we deal with a splitting peak (Figure S1). Succinic acid (**1**), which is less hydrophilic than acids **2**–**4**, is eluted later than **2**–**4**, and with minor tailing (Figure S1). Therefore, for compounds **2**–**4,** the hydrophilic interaction chromatography would be more appropriate [27,28,29]. On the other hand, for the purpose of this work (LC-MS analysis of their monoethyl esters), the use of reversed-phase liquid chromatography is justified. Acids **1**–**4** are very common compounds, having been reported on for years, and the fragmentation pathways of their [M − H]^−^ ions can be easily deduced from the database mass spectra (https://massbank.eu/MassBank/, accessed on 10 September 2026).

Phenolic acids **5**–**7** are quite common in wines, including Tokaji wines [22,23], and the product ions coming from their [M − H]^−^ ions have already been reported [19,25,30]. The ESI mass spectra of **5** and **6** (the abundances of **7** were too low to obtain pure mass spectra) obtained at a cone voltage of 50 V, shown in Figure S3, are in acceptable agreement with the published data [19,25,30].

### 2.2. LC-MS Identification of Monoethyl Esters of Aliphatic Acids

For acids **2**–**4**, monoethyl esters (**2e**–**4e**) were detected, and for citric acid **4**, besides monoethyl ester **4e** at *m*/z 219, diethyl ester **4ee** was also detected at *m*/*z* 247, although its abundance was ~10-fold lower than that of **4e** (Supplementary Material, Figure S4). It is surprising that monoethyl ester of succinic acid (**1**) was not detected (the signal at *m*/*z* 145 had very low intensity). It had most likely been esterified to the diethyl succinate [17], which cannot be detected in the negative ion mode. Monoethyl esters **2e**–**4e** are not particularly common and, to the best of our knowledge, the fragmentation pathways of their [M − H]^−^ ions have not been studied.

**Figure 1:**
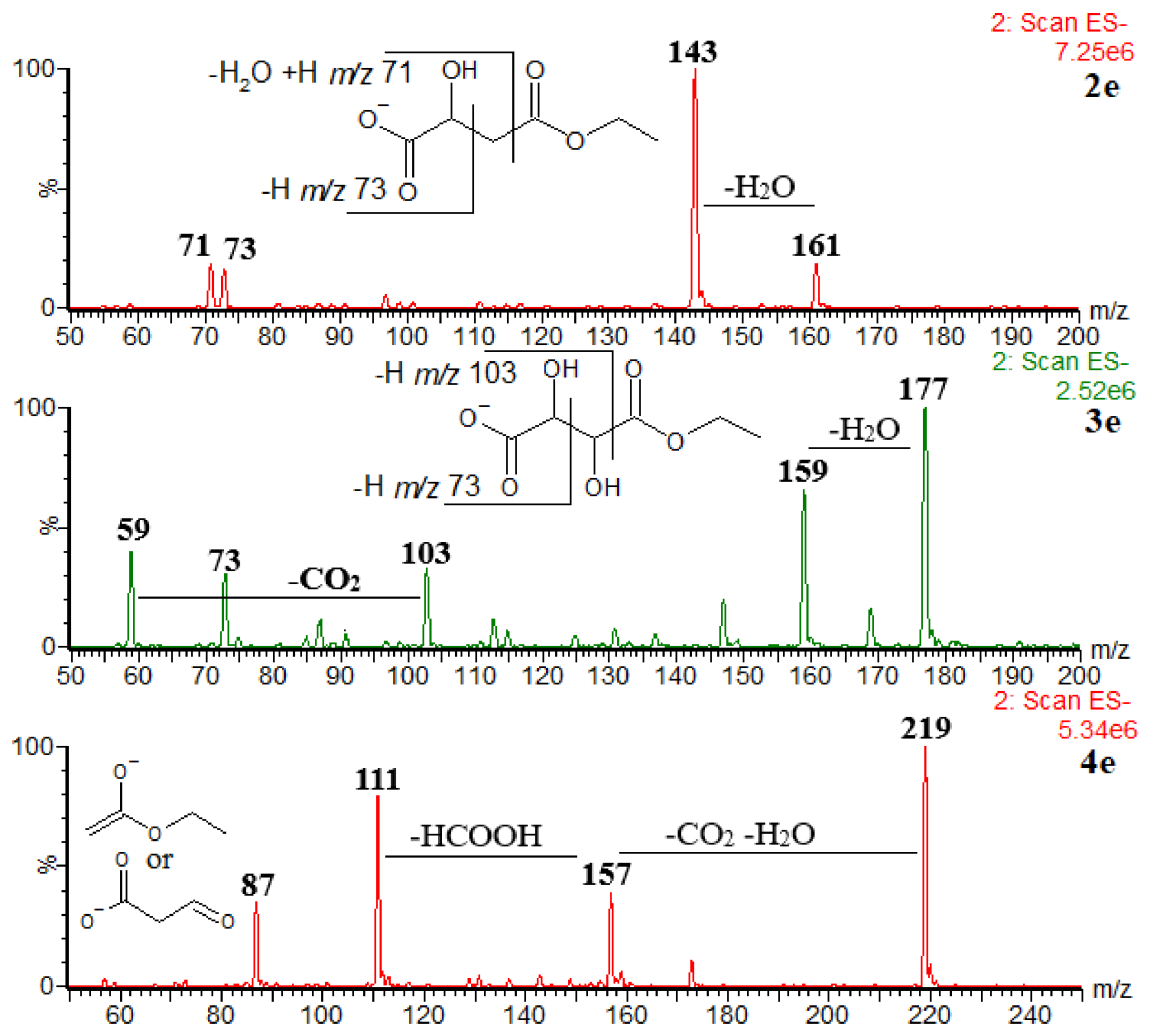
ESI mass spectra of **2e**, **3e**, and **4e** obtained at a cone voltage of 50 V.

It is expected that ester **3e** (monoethyl tartrate) occurs in two diastereomeric isomers in comparable abundance, which was confirmed by the shape of its chromatographic peak (Figure S4). For both isomers, practically identical ESI mass spectra were obtained (Figure 1). The loss of H_2_O is also dominant (*m*/*z* 159), and the other product ions (*m*/*z* 103, 73, 59) are moderately abundant.

Esters **2e** and **4e** can occur in two isomers which differ in the ethyl group substitution to different carboxylic groups. The shape of the **2e** (monoethyl malate) peak indicated with the presence of one of the possible isomers. On the basis of the fragmentation pattern of **2e**, the structure of this isomer was deduced (Figure 1). Although the dominant product ion is [M − H − H_2_O]^−^ (which is uninformative for structure determination), the product ion at *m*/*z* 71 and 73 (less abundant but clearly seen) enables the determination of the site of the ethyl group substitution (Figure 1). These product ions cannot be expected to be present within the second possible isomer.

The shape of **4e** (monoethyl citrate) peak indicated that we deal with the two possible isomers, one of which is dominant (Figure S4). For both isomers, practically identical ESI mass spectra were obtained (Figure 1). We dealt with the losses of H_2_O, CO_2_, and HCOOH molecules (*m*/*z* 157 and 111). A product ion was also identified at *m*/*z* 87 in moderate abundance, for which two plausible structures can be proposed (Figure 1). Therefore, on the basis of [M − H]^−^ ions fragmentation, it was not possible to determine which of the possible isomers was dominant. All detected product ions can be expected to be located within both possible isomers of **4e**. As described later on, their differentiation was proven possible in the positive ion mode.

### 2.3. LC-MS Identification of Monoethyl Esters of Phenolic Acids

**Figure 2:**
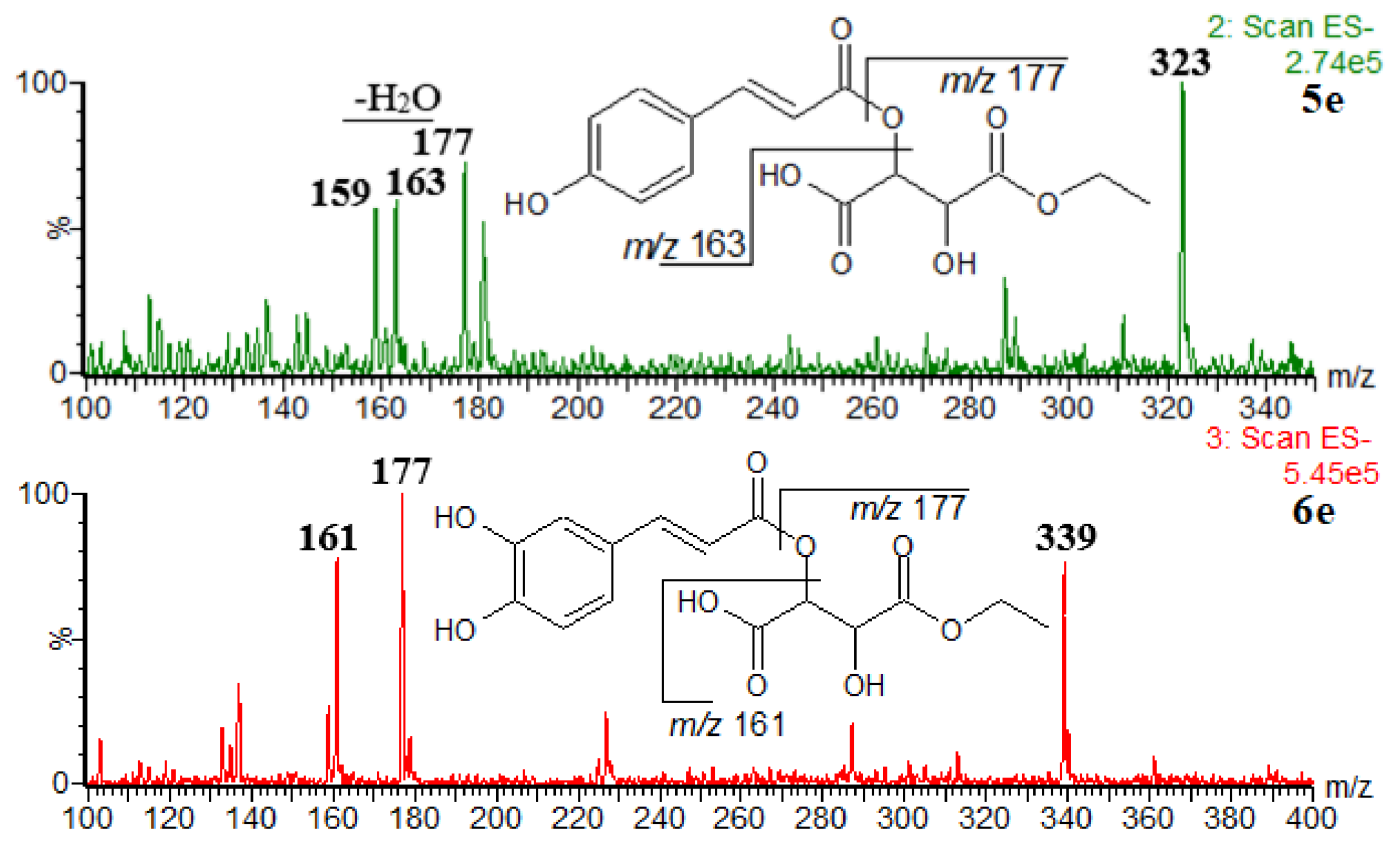
ESI mass spectra of **5e** and **6e** obtained at a cone voltage of 50 V.

Esters **5e**–**7e** are very rare and, to the best of our knowledge, there are only three reports on their detection in wine [24,25,26], not including Tokaji wines. It is surprising that for such common acids (**5**–**7**) occurring in wines, there are so few reports on their detection. Exemplary single-ion chromatograms of [M − H]^−^ are shown in the Supplementary Material (Figure S5). The ESI mass spectra of **5e** and **6e** (the abundances of **7e** were too low to obtain a pure mass spectrum) recorded at a cone voltage of 50 V are shown in Figure 2. These findings are in agreement with the published data (although the published data have not reported these findings in such detail) [25,26]. For both esters, we deal with the breaking of ester bonds, and the dominant product ion is that found at *m*/*z* 177, which formally corresponds to the deprotonated ethyl tartrate.

### 2.4. LC-MS Identification of Other Compounds

Besides the above-mentioned compounds, two other compounds, namely ethyl caffeate (quite common monoethyl ester) and ascorbic acid, were identified in all the analyzed samples (Figure S6). In contrast to all other esters, the acid of ethyl caffeate (caffeic acid) was not detected. This situation is not very surprising because such cases have already been noted in wine analysis [18,24,25]. Ascorbic acid is present in grapes; however, the amount is decreased during the wine production process [13,31,32]. The high relative amounts of ascorbic acid in the analyzed samples are most probably due to its addition to the wines as a complementary/alternative antioxidant preservative to sulfur dioxide, although the benefits of ascorbic acid addition may be disputable [33,34].

### 2.5. LC-QTOF-MS/MS Analysis in the Positive Ion Mode

**Figure 3:**
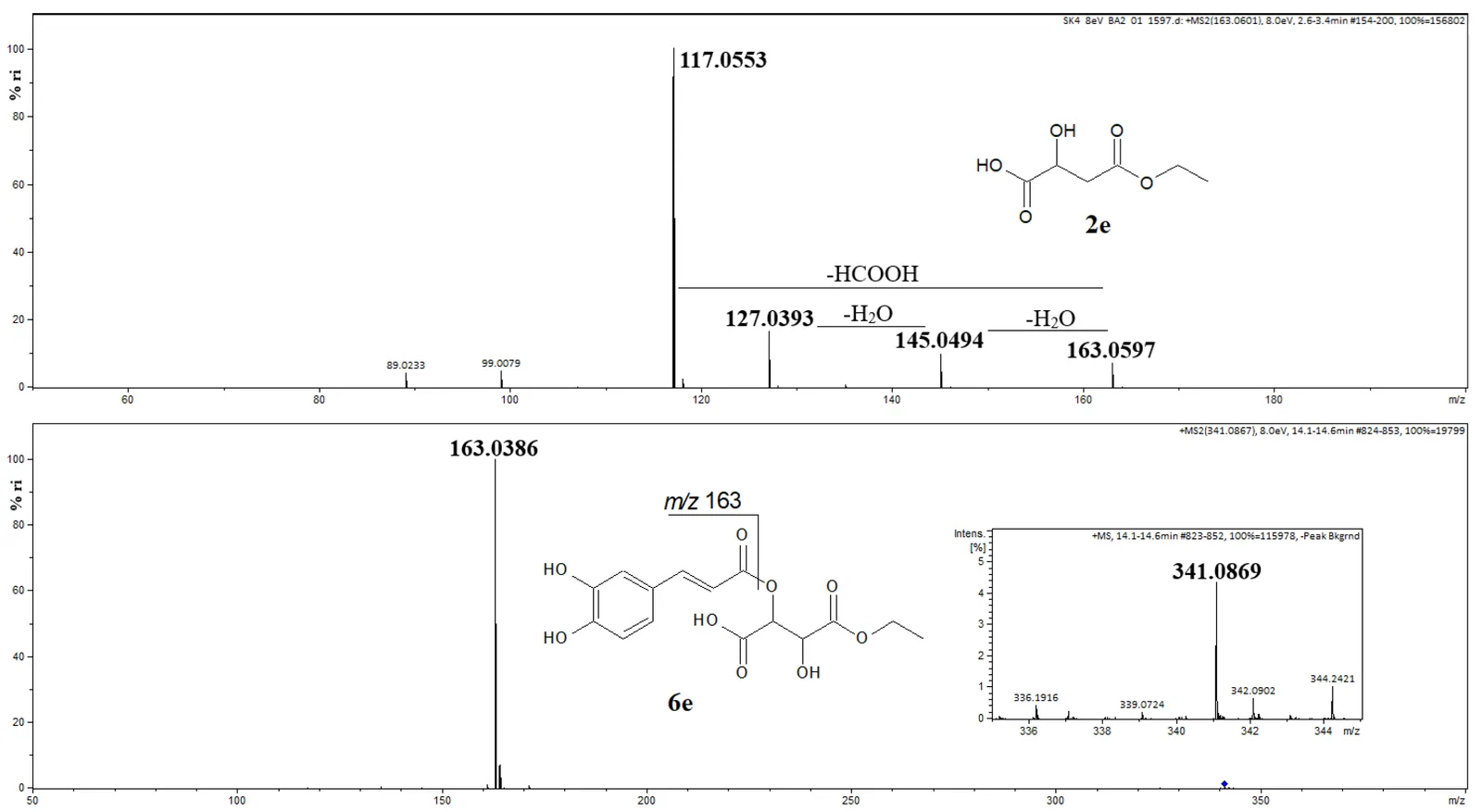
Product ion spectra of the [M + H]^+^ ions from **2e** (*m*/*z* 163) and **6e** (*m*/*z* 341).

The compounds analyzed under LC-MS conditions yielded very low signals in the positive ion mode, especially in the full scan mode. In order to further confirm the structures of the analyzed rare esters, we performed LC-MS analysis in the MS/MS mode using a QTOF mass spectrometer. This enabled us to obtain the accurate *m*/*z* of the [M + H]^+^ ions in order to confirm their elemental composition, as well as to obtain the product ion spectra, so as to determine the fragmentation pathways of the analyzed compounds in the positive ion mode. Figure 3 shows the product ion spectra of **2e** and **6e** obtained in the positive ion mode as representative examples. The fragmentation of aliphatic ester **2e** involves the loss of small molecules (H_2_O and HCOOH), and the fragmentation of phenolic ester **6e** involves only the formation of abundant product ions at *m*/*z* 163 (Figure 3).

**Figure 4:**
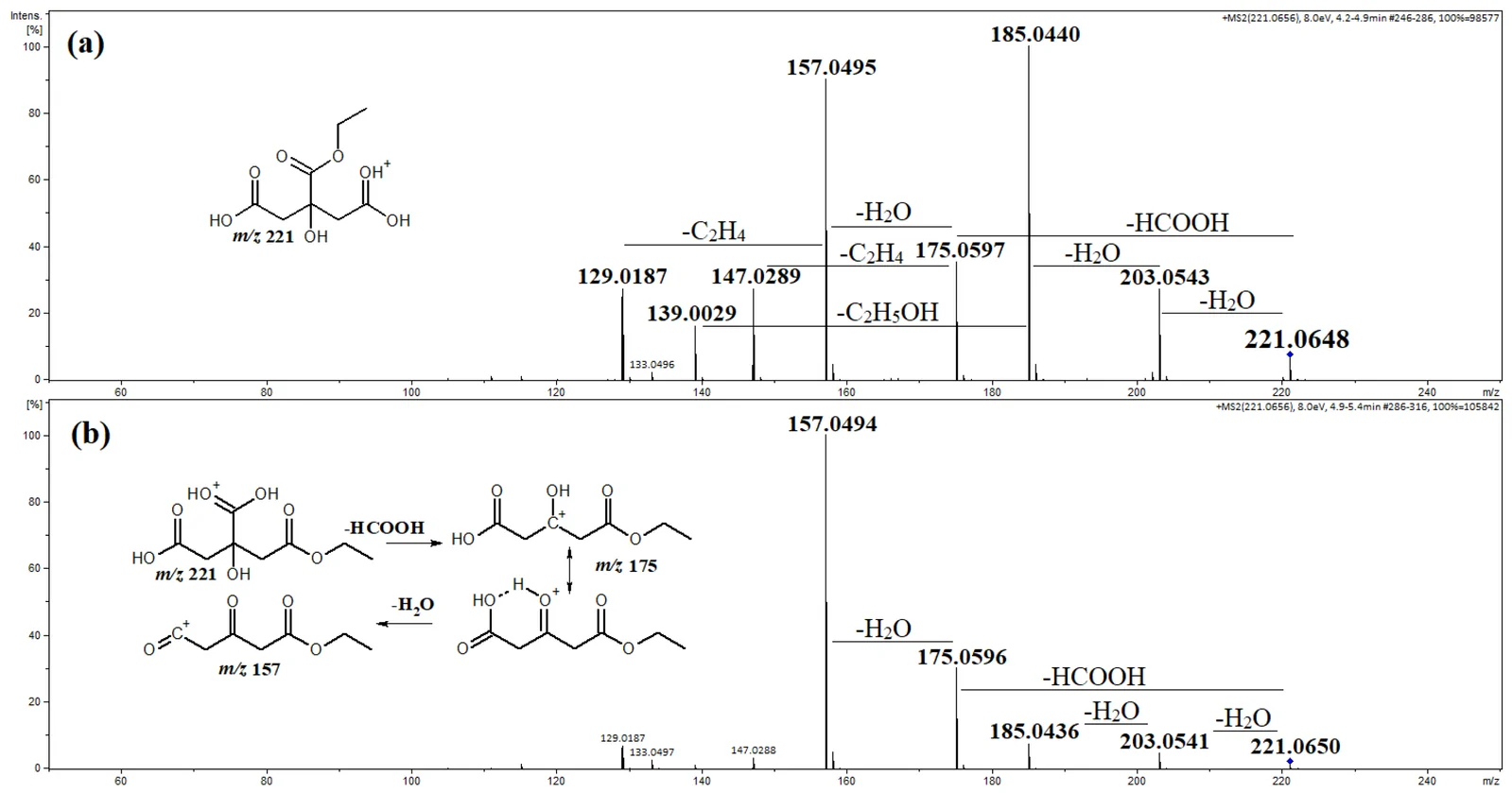
Product ion spectra of the [M + H]^+^ ions from monoethyl citrate (**4e**) isomers (**4a** and **4b**).

It was found that the product ion spectra of the isomers of monoethyl citrate (**4e**) differed substantially in the relative abundances of product ions (Figure 4). The fragmentation pathway of the [M + H]^+^ ion of the first isomer (less abundant, eluted as the first one) consists of consecutive losses of small molecules (H_2_O, HCOOH, C_2_H_4_, C_2_H_5_OH) and is very complicated (Figure 4a). However, the fragmentation pathway of the [M + H]^+^ ion of the second isomer (more abundant, eluted as the second one) consists mainly of the loss of HCOOH (*m*/*z* 175), followed be the loss of H_2_O (*m*/*z* 157, Figure 4b).

It is reasonable that this fragmentation pathway is favored for the isomer having a free carboxyl group substituted to the central carbon atom. The loss of HCOOH molecule yields a product ion at *m*/*z* 175, which can be stabilized by resonance (the stabilization which is not possible for the first isomer), and then due to the intramolecular hydrogen bond the H_2_O loss yields a product ion at *m*/*z* 175, as shown in Figure 4b.

### 2.6. Comparative Analysis of the Abundances of the Analyzed Compounds in Wine Samples

In order to compare the relative amounts of the identified esters in the wine samples, the areas of the chromatographic peaks of [M − H]^−^ ions obtained at the cone voltage of 30 V were compared. We are aware of the fact that a comparison of [M − H]^−^ ion abundances reflects only semi-quantitatively the relative amounts of the esters in the wine sample. On the other hand, it is reasonable for the ESI responses of **2e**–**4e**, and **5e**–**7e** to be comparable, since they are groups of compounds of the same type (aliphatic and phenolic acids, respectively), and their ionization in the negative ion mode involves the deprotonation of the carboxyl group. It is known that the signal response obtained after some time (in identical instrumental conditions) may be different because of the fluctuations in pressure inside the mass spectrometer [35,36]. Therefore, the comparison of the peak areas between the samples was based on the results of the analyses performed in immediate subsequence. Furthermore, in order to check if the peak areas are proportional to the amount of the compounds introduced to the LC-MS system, LC-MS analyses were performed by using a number of injection volumes (1–30 μL). The Supplementary Material (Figure S7) presents representative plots. The obtained relationships between injection volumes and peak areas are acceptable (R^2^ > 0.9).

**Figure 5:**
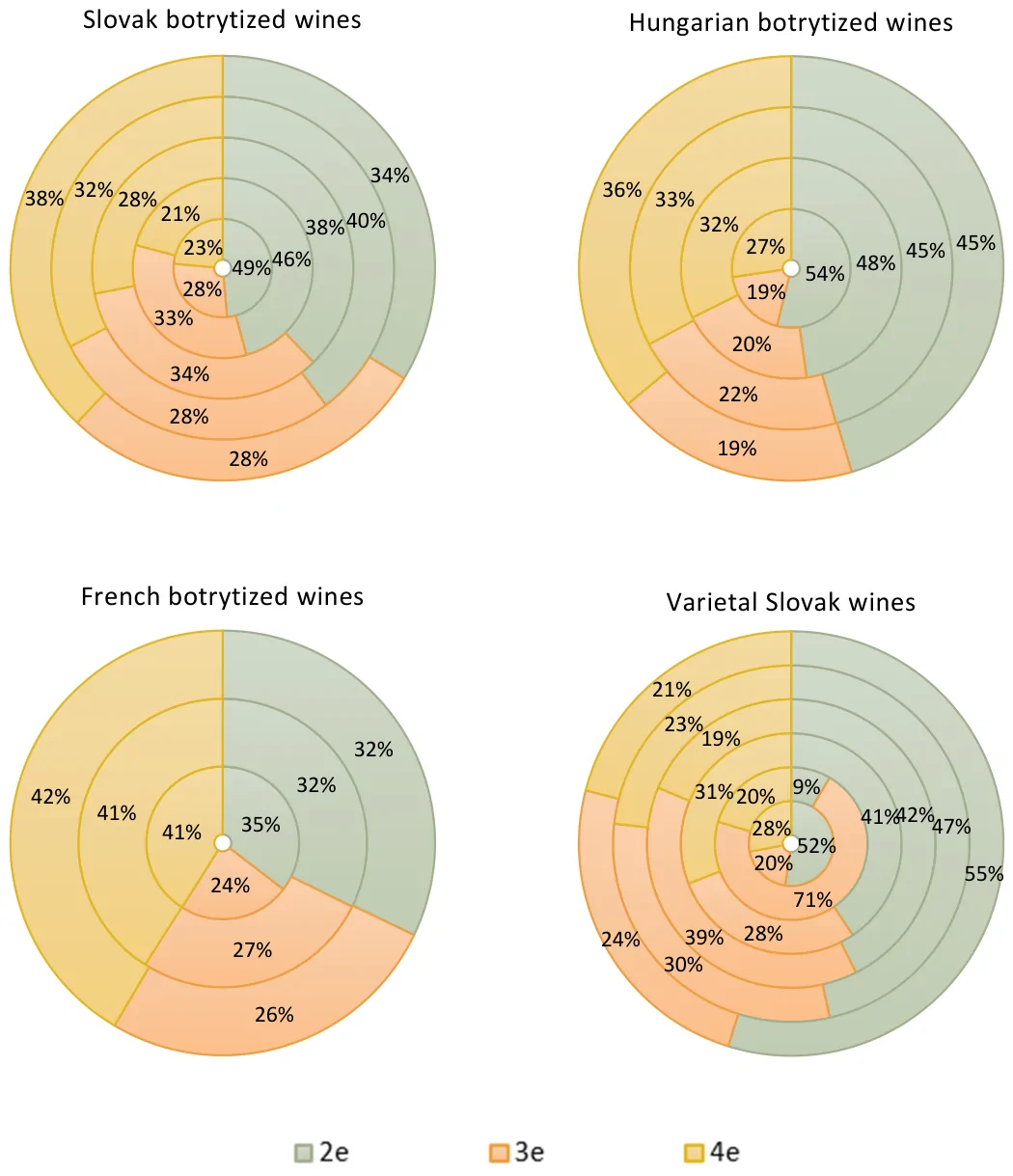
A comparison of the relative chromatographic peak areas of [M − H]^−^ ions of **2e**–**4e** obtained for the analyzed wine samples. The concentric circles, arranged from the innermost to the outermost ring, correspond to individual samples: e.g., for botrytized Slovak wines (**SK_A–E**), the innermost ring corresponds to the sample **SK_A**, whereas the outermost ring corresponds to the sample **SK_E**; for botrytized Hungarian wines (**HU_A–D**), the innermost ring corresponds to the sample **HU_A**, whereas the outermost ring corresponds to the sample **HU_D**; the same is valid for botrytized French wines (**FR_A–C**) and varietal wines (**SK_F–K**).

Figure 5 shows a comparison of the chromatographic peak areas of the [M − H]^−^ ions of **2e**–**5e** (aliphatic esters) obtained from the analyzed wine samples. The values obtained for Hungarian botrytized wines (samples **HU_A–D**) and for the French botrytized wines (samples **FR_A–C**) were the most similar, although the wines analyzed are of different vintages (Table 1).

The Hungarian botrytized wines show very similar relative amount of **3e** (19–22%), and the French botrytized wines show similar relative amounts of all aliphatic esters (the differences between the samples exceed 3%). The relative amounts of **2e**–**5e** in Slovak botrytized wines (samples **SK_A–E**) and varietal wines produced in the Tokaj region (**SK_F–K**) differ between these samples and also do not resemble the values obtained for Hungarian and French botrytized wines (Figure 5).

**Figure 6:**
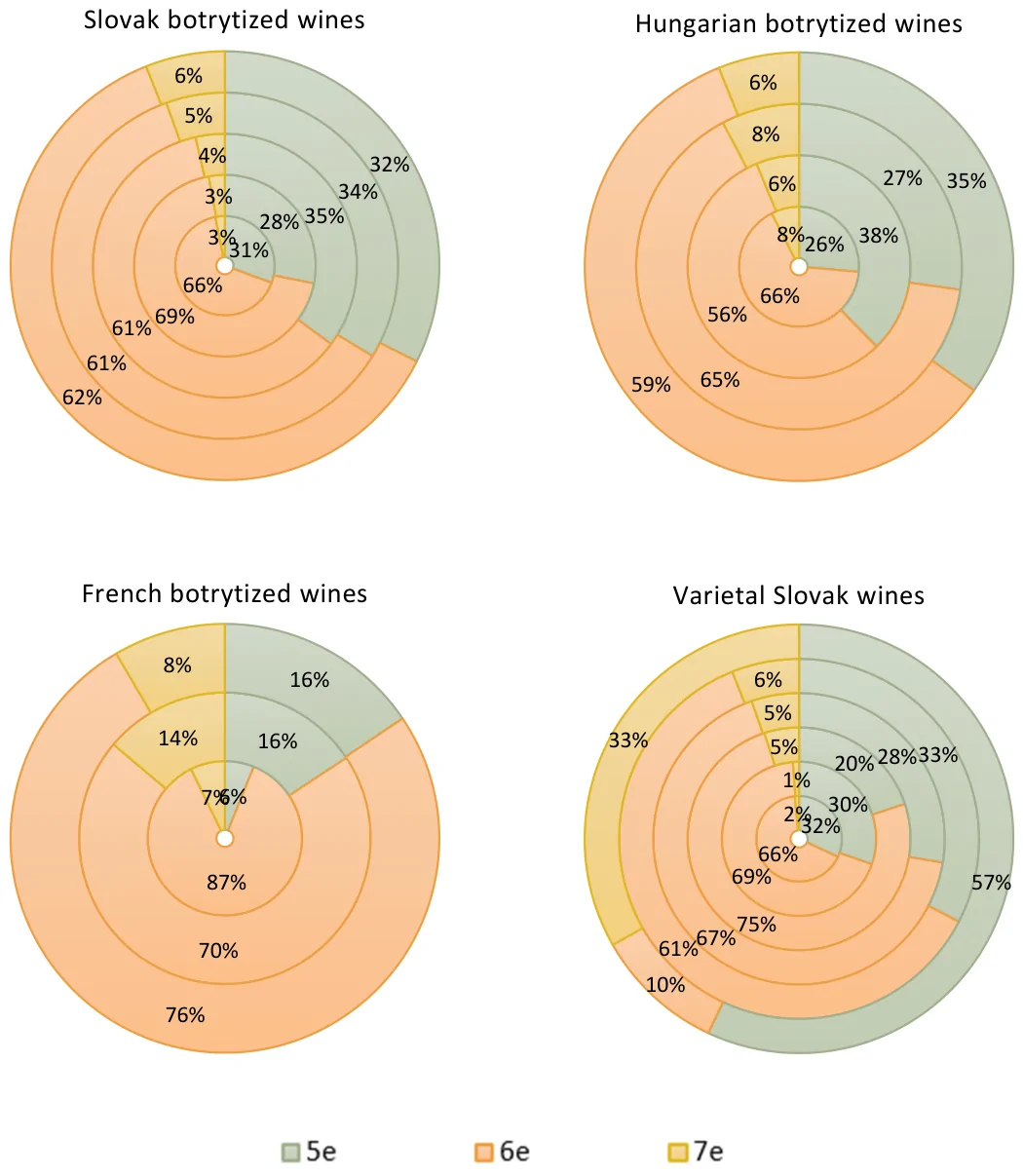
A comparison of the relative chromatographic peak areas of [M − H]^−^ ions of **5e**–**7e** obtained for the analyzed wine samples. The concentric circles, arranged from the innermost to the outermost ring, correspond to individual samples: e.g., for botrytized Slovak wines (**SK_A–E**), the innermost ring corresponds to the sample **SK_A**, whereas the outermost ring corresponds to the sample **SK_E**; for botrytized Hungarian wines (**HU_A–D**), the innermost ring corresponds to the sample **HU_A**, whereas the outermost ring corresponds to the sample **HU_D**; the same is valid for botrytized French wines (**FR_A–C**) and varietal wines (**SK_F–K**).

Figure 6 shows a comparison of the chromatographic peak areas of [M − H]^−^ ions of **5e**–**7e** (phenolic esters) obtained for the analyzed wine samples. The most similar were found to be the values obtained for Slovak botrytized wines (samples **SK_A–E**). The relative amount of **6e** is in the range 61–69%. The Hungarian botrytized wines (samples **HU_A–D**) show very similar relative amount of **7e** (6–8%). It is worth mentioning that wines **SK_A–E** and **HU_A–D** are of different vintages (Table 1).

The relative amounts of **5e**–**7e** in French botrytized wines and varietal wines produced in the Tokaj region differ between these samples and also do not resemble the values obtained for the Slovak and Hungarian botrytized wines (Figure 6).

There is an increasing need to develop simple and quick methods for reliable assessment of the quality of high-quality wines in view of the high risk of adulteration [37,38,39,40]. A few methods have been proposed for the reliable assessment of the quality of Tokaj wines, namely those based on UV spectroscopy, IR spectroscopy, chemometrics, and electronic tongue [41,42,43,44]. Similar relative amounts of some of the identified unique esters in botrytized wines (even though they came from different vintages) offer the potential to use these compounds as authenticity markers for this type of wine, although we are aware that this conclusion needs to be confirmed using isotopically labeled standards.

## 3. Materials and Methods

### 3.1. Wine Samples

The wine samples are described in Table 1 and Table 2. The selected wines are aged wines, and their selection was dictated by market availability. Tokaj wine production in the Tokaj wine region of Hungary and Slovakia is based on a traditional unit of measurement called putňa (puttony). This refers to a basket (approximately 20–25 kg) of botrytized berries added to a 136 L barrel of grape must. Tokaj wines are marked with a puttony number of 3, 4, 5, or 6 to indicate the wine’s sweetness, flavor, and concentration—formerly based on the number of baskets used, now primarily on sugar content [21]. The botrytised wines are commonly aged for at least one year. For example, the oxidative aging of Slovak Tokaj wines takes place in small 136 L oak barrels and generally lasts 3–5 years [11]. The botrytised wines are stored in the fridge, whereas the varietal wines are stored in the freezer after the bottles are opened. The pH of the analyzed wines varied from 3 to 4; all other information (e.g., alcohol and sulphite content) is provided in Table S1.

### 3.2. LC-MS Analysis of Wine Samples

Before the LC-MS analysis, the wine samples (stored at room temperature) were filtered through syringe filters with a pore size of 0.45 μm (Macherey-Nagel GmbH, Düren, Germany). No other sample preparation was performed. LC-MS analyses were carried out using a Waters Arc HPLC pump and a Waters SQD mass spectrometer (single quadrupole-type instrument equipped with electrospray ionization (ESI) source, Z-spray, Milford, MA, USA). The software used was MassLynx V4.2 SCN1046 (Milford, MA, USA). The data obtained by this software were processed and analyzed using the widely available Excel program. The sample solutions were injected into Atlantis C18 T3 column (3 μm, 100 mm × 3 mm i.d., Waters, Warsaw, Poland) using an autosampler. The solutions were analyzed using a linear gradient of CH_3_CN-H_2_O (both solvents were obtained from Merck (suitable for LC-MS, LiChrosolv^®^), Poznań, Poland) grade, at a flow rate of 0.5 mL/min. The gradient started from 0% CH_3_CN and 95% H_2_O with 5% of a 10% solution of formic acid in water, reaching 95% CH_3_CN (and 0% H_2_O) after 15 min, and the latter concentration was kept for 10 min. The LC-MS was performed in the negative ion mode in the *m*/*z* range 100–1000 or 50–500. Each sample was analyzed three times, and the calculated relative standard deviation of peak areas did not exceed 7%. The nebulizing and desolvation gases were both nitrogen at flow rates of 100 and 300 L/h, respectively. The source temperature was 120 °C, and the desolvation temperature was 300 °C. The electrospray source potentials were capillary 3 kV, lens 0.5 V, and extractor 4 V, with cone voltages (CVs) of 30 V and 50 V. This parameter has the greatest impact on the full scan mass spectra recorded. An increase in this parameter can lead to a so-called “in-source” fragmentation/dissociation, but a too-low cone voltage may cause a decrease in sensitivity.

It should be stressed that the retention times of parent and product ions were carefully checked, and they always matched perfectly; therefore, there are no doubts that the product ions are really coming from the respective [M − H]^−^ ion.

### 3.3. LC-QTOF-MS/MS Analysis

These experiments were performed using an UltiMateTM 3000 UHPLC system (ThermoScientific/Dionex, Sunnyvale, CA, USA) and an Impact HD mass spectrometer (QTOF type instrument equipped with electrospray ion source; Bruker Daltonics, Billerica, MA, USA). Using an autosampler, the sample solutions were injected into the Kinetex C18 column (100 × 2.10 mm i.d., 2.6 μm particle size). The mobile phases used were water with 0.1% formic acid (solvent A) and acetonitrile with 0.1% formic acid (solvent B). The samples were analyzed using a linear gradient at a flow rate of 0.3 mL/min and the column temperature was maintained at 35 °C. The gradient was programmed as follows: 90% A and 10% B for 1 min, followed by a linear change to 5% A and 95% B in 20 min, held for 10 min. The instrument was operated in the positive ion mode, under the following optimized settings: end-plate voltage 500 V, capillary voltage 4.2 kV; collision energy 8 eV, nebulizer pressure 1.5 bar; dry gas (nitrogen); temperature 200 °C; dry gas flow rate 8 L/min. The spectrometer was previously calibrated with the standard tune mixture. Although the applied collision energy was low, namely 8 eV, the selected parent ions [M + H]^+^ ions were of very low abundances in comparison to those of the product ions.

## 4. Conclusions

In all wine samples analyzed by the LC-MS, six rare monoethyl esters were detected: three aliphatic and three phenolic. Their structures, including isomer differentiation of monoethyl malate and monoethyl citrate, were deduced on the basis of characteristic fragmentation pathways of [M − H]^−^ and [M + H]^+^ ions. It was found that the relative abundances of some the detected unique compounds are very similar for botrytized wines, in contrast to the varietal Slovak wines, e.g., the relative amount of monoethyl caftarate (**6e**) in Slovak botrytized wines was in the range of 61–69%, and the Hungarian botrytized wines showed very similar relative amounts of monoethyl fertarate (**7e**), namely 6–8%. The observed similarities may be attributable to the botrytization process and also to the specific aging and storage conditions. The results offer the potential to use these unique compounds as authenticity markers for botrytized wines.

## Figures and Tables

**Table 1:** A list of the botrytized wines.

Country	Vintage	Type (Putňa)	Manufacturer	Code
Slovakia	2011	3	Tokaj & CO (Malá Tŕňa, Slovakia)	**SK_A**
Slovakia	2006	6	Tokaj & CO (Malá Tŕňa, Slovakia)	**SK_B**
Slovakia	2014	3	Zlatý Strapec (Viničky, Slovakia)	**SK_C**
Slovakia	2013	5	Zlatý Strapec (Viničky, Slovakia)	**SK_D**
Slovakia	2003	3	J&J Ostrožovič (Veľká Tŕňa, Slovakia)	**SK_E**
Hungary	2008	5	Sajgó Pincészet (Tolcsva, Hungary)	**HU_A**
Hungary	2013	6	Sajgó Pincészet (Tolcsva, Hungary)	**HU_B**
Hungary	2013	4	Simkó Pince (Sárospatak, Hungary)	**HU_C**
Hungary	2014	6	Simkó Pince (Sárospatak, Hungary)	**HU_D**
France	2011	Sauternes	Château Rieussec (Fargues-de-Langon, France)	**FR_A**
France	1995	Sauternes	Château d’Yquem (Sauternes, France)	**FR_B**
France	1999	Sauternes	Château Suduiraut (Preignac, France)	**FR_C**

**Table 2:** A list of the varietal wines produced in the Tokaj wine region.

Country	Vintage	Type	Manufacturer	Code
Slovakia	2010	Yellow Muscat	J&J Ostrožovič (Veľká Tŕňa, Slovakia)	**SK_F**
Slovakia	2007	Yellow Muscat	J&J Ostrožovič (Veľká Tŕňa, Slovakia)	**SK_G**
Slovakia	2013	Furmint	J&J Ostrožovič (Veľká Tŕňa, Slovakia)	**SK_H**
Slovakia	2012	Furmint	Tokaj Macik Winery (Malá Tŕňa, Slovakia)	**SK_I**
Slovakia	2013	Lipovina	J&J Ostrožovič (Veľká Tŕňa, Slovakia)	**SK_J**
Slovakia	2010	Lipovina	Tokaj Macik Winery (Malá Tŕňa, Slovakia)	**SK_K**

## Data Availability

The original contributions presented in this study are included in the article. Further inquiries can be directed to the authors (O.S. and M.F.).

## Supplementary Materials

The following supporting information can be downloaded at: https://www.mdpi.com/article/10.3390/molecules31203776/s1, Figure S1: Single-ion chromatograms of [M − H]^−^ ions of succinic (**1**), malic (**2**), tartaric (**3**), and citric (**4**) acids; Figure S2: Single-ion chromatograms of [M − H]^−^ ions of coutaric (**5**), caftaric (**6**), and fertaric (**7**) acids; Figure S3: ESI mass spectra of **5** and **6** obtained at a cone voltage of 50 V; Figure S4: Single-ion chromatograms of [M − H]^−^ ions of monoethyl malate (**2e**), monoethyl tartrate (**3e**), monoethyl citrate (**4e**), diethyl citrate (**4ee**); Figure S5: Single-ion chromatograms of [M − H]^−^ ions of phenolic esters; coumaroyl ethyl tartrate (**5e**, monoethyl coutarate), caffeoyl ethyl tartrate (**6e**, monoethyl caftarate), feruloyl ethyl tartrate (**7e**, monoethyl fertarate); Figure S6: Single-ion chromatograms of [M − H]^−^ ions of ethyl caffeate (**8**) and ascorbic acid (**9**); Figure S7: Exemplary breakdown plots of chromatographic peak areas (arbitrary units) against injection volume; Table S1: Information concerning alcohol and sulphite content and other information provided by the manufacturer.
