# Supplementary material for: LC-MS Identification of Rare Ethyl Esters in Botrytized Wines
Source: Molecules. 2026 Oct 21;31(20):3776. doi: 10.3390/molecules31203776 (PMC13707519; doi:10.3390/molecules31203776)
Supplement: Supplementary file 1 [file molecules-31-03776-s001.zip › molecules-4592082-supplementary.pdf]

LC-MS Identification of Rare Ethyl Esters in Botrytized Wines

SUPPLEMENTARY MATERIAL

Olga Stężycka, Daniel Górny, Olga Vyviurska, Ivan Špánik, Małgorzata Kasperkowiak and Magdalena Frańska

Table of contents:

Figure S1. Single ion chromatograms of $[M-H]^-$ ions of succinic (**1**), malic (**2**), tartaric (**3**), and citric (**4**) acids. 2

Figure S2. Single ion chromatograms of $[M-H]^-$ ions of coutaric (**5**), caftaric (**6**), and fertaric (**7**) acids. 2

Figure S3. ESI mass spectra of **5** and **6** obtained at a cone voltage of 50 V. 3

Figure S4. Single ion chromatograms of $[M-H]^-$ ions of monoethyl malate (**2e**), monoethyl tartrate (**3e**), monoethyl citrate (**4e**), diethyl citrate (**4ee**). 3

Figure S5. Single ion chromatograms of $[M-H]^-$ ions of phenolic esters; coumaroyl ethyl tartrate (**5e**, monoethyl coutarate), caffeoyl ethyl tartrate (**6e**, monoethyl caftarate), feruloyl ethyl tartrate (**7e**, monoethyl fertarate). 4

Figure S6. Single ion chromatograms of $[M-H]^-$ ions of ethyl caffeate (**8**) and ascorbic acid (**9**). 4

Figure S7. Exemplary breakdown plots of chromatographic peak areas (arbitrary units) against injection volume. 5

Table S1. Information concerning alcohol and sulphites content and other information provided by manufacturer. 6

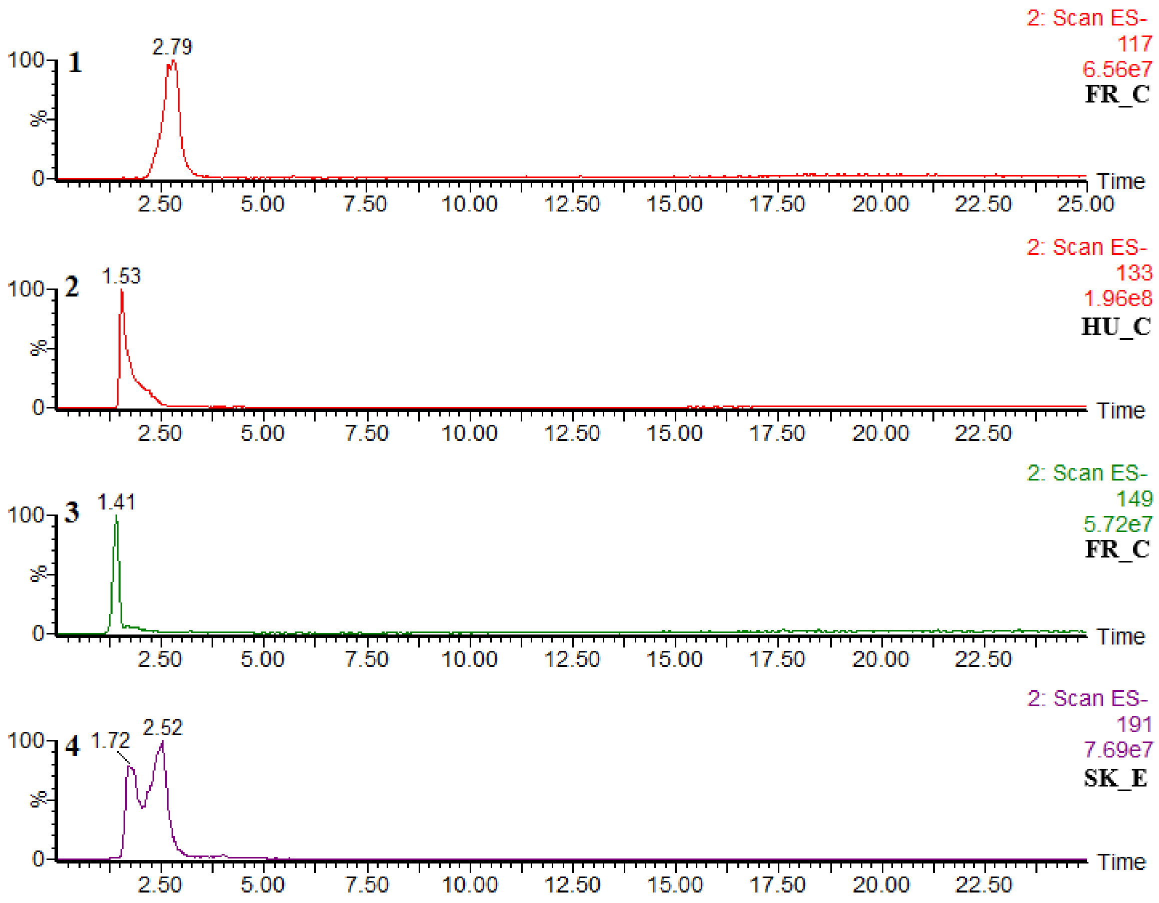

Figure S1. Single ion chromatograms of $[M{-}H]^-$ ions of succinic (**1**), malic (**2**), tartaric (**3**), and citric (**4**) acids.

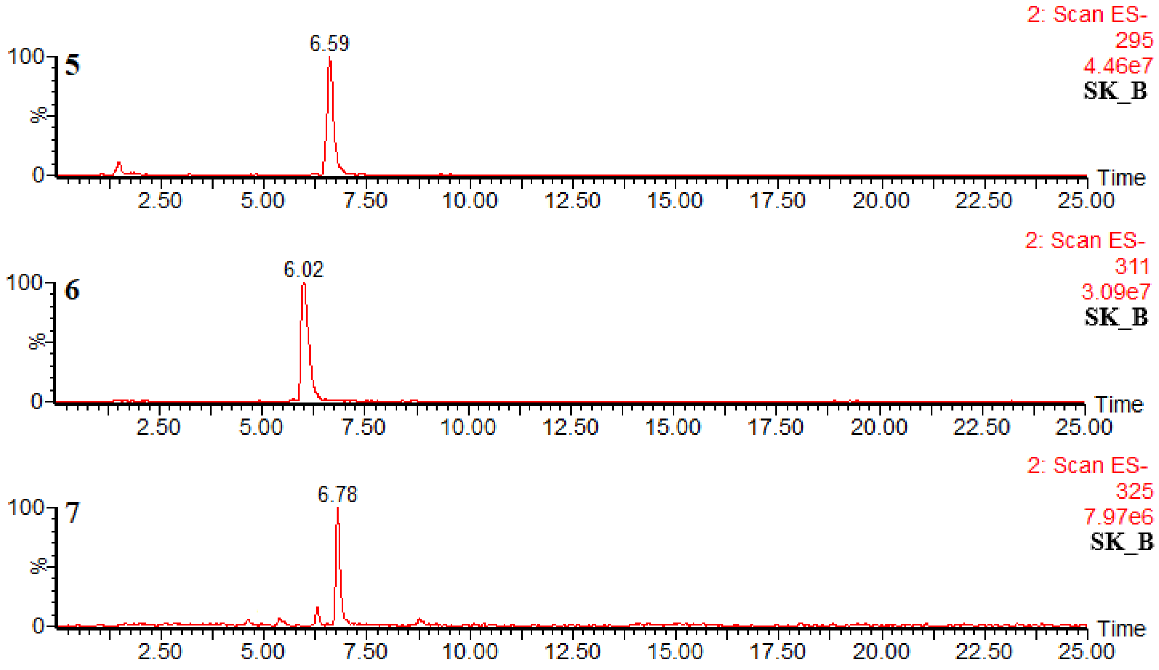

Figure S2. Single ion chromatograms of $[M{-}H]^-$ ions of coutaric (**5**), caftaric (**6**), and fertaric (**7**) acids.

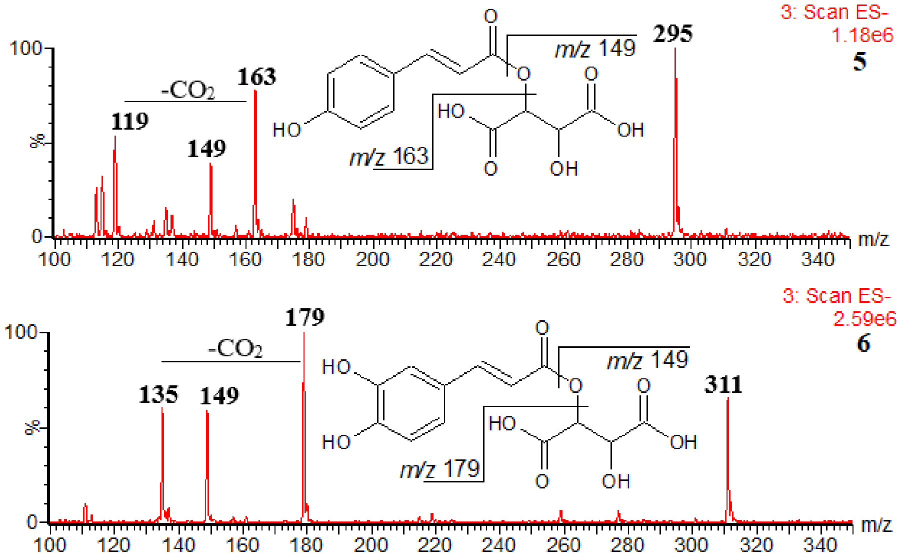

Figure S3. ESI mass spectra of **5** and **6** obtained at a cone voltage of 50 V.

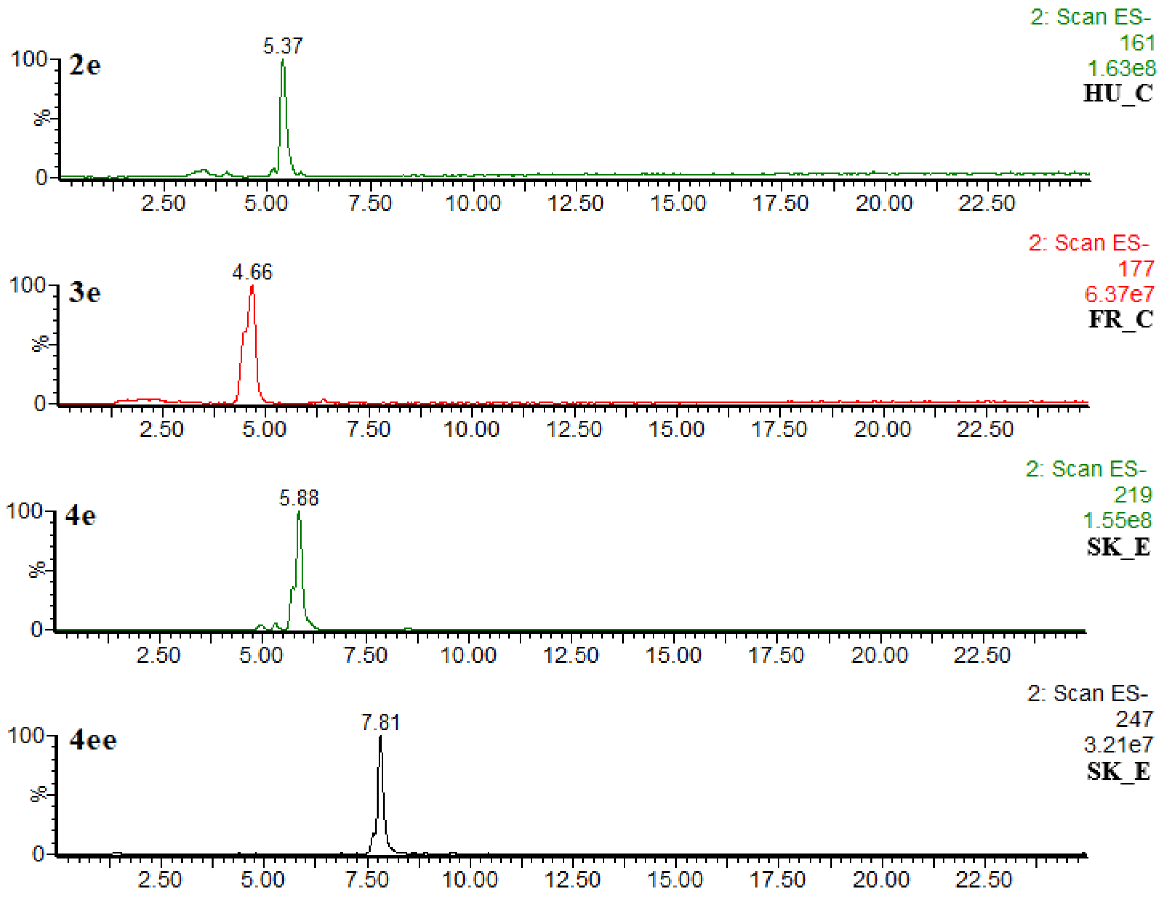

Figure S4. Single ion chromatograms of $[M-H]^-$ ions of monoethyl malate (**2e**), monoethyl tartrate (**3e**), monoethyl citrate (**4e**), diethyl citrate (**4ee**).

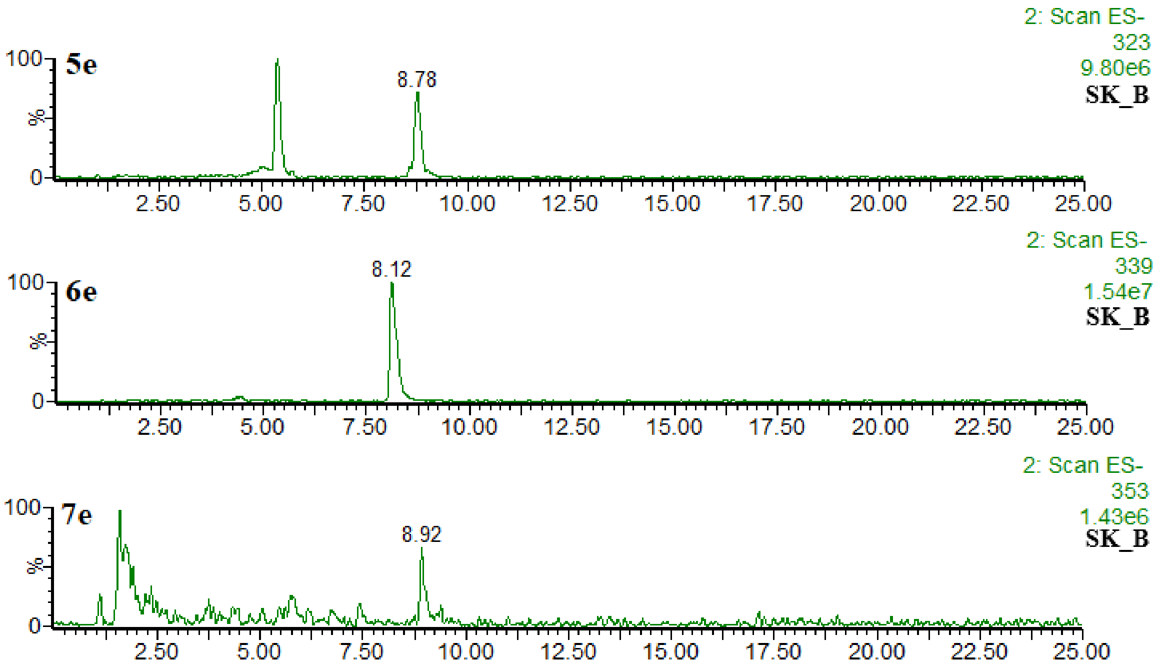

Figure S5. Single ion chromatograms of [M−H]$^{-}$ ions of phenolic esters; coumaroyl ethyl tartrate (**5e**, monoethyl coutarate), caffeoyl ethyl tartrate (**6e**, monoethyl caftarate), feruloyl ethyl tartrate (**7e**, monoethyl fertarate).

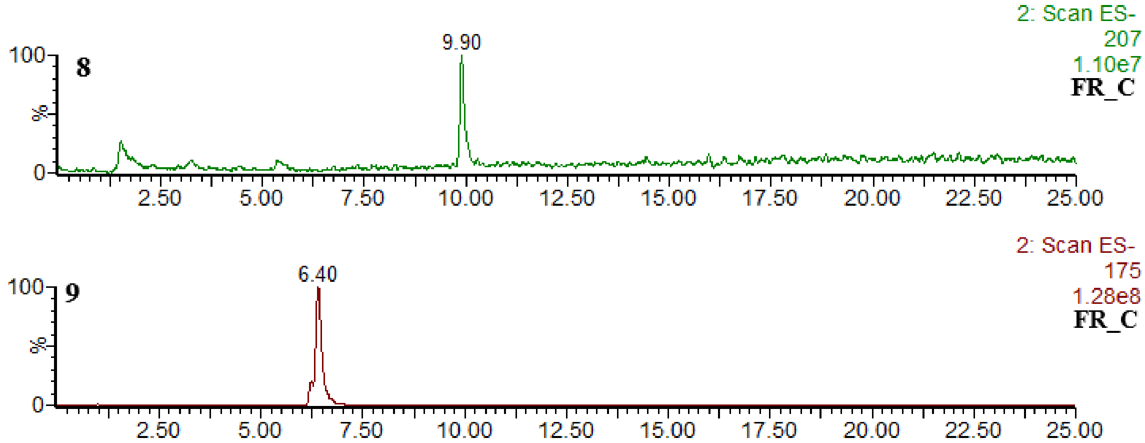

Figure S6. Single ion chromatograms of [M−H]$^{-}$ ions of ethyl caffeate (**8**) and ascorbic acid (**9**).

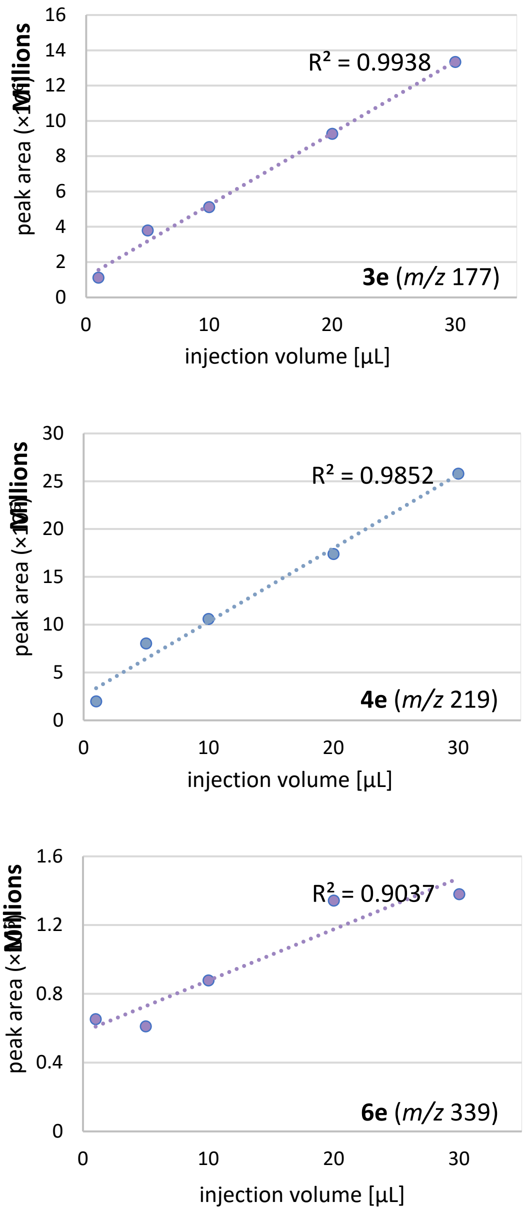

Figure S7. Exemplary breakdown plots of chromatographic peak areas (arbitrary units) against injection volume.

Table S1. Information concerning alcohol and sulphites content and other information provided by manufacturer.

Sample code	Alcohol content [% V]	Information provided on the label
**HU_A**	13,5	contains sulphites
**HU_B**	11,5	contains sulphites
**HU_C**	11,5	contains sulphites
**HU_D**	11,5	contains sulphites
**SK_A**	11	contains sulfur dioxide/ sulfur monooxide
**SK_B**	10	contains sulfur dioxide/ sulfur monooxide
**SK_C**	12,5	contains sulphites
**SK_D**	12,5	contains sulphites
**SK_E**	12	contains sulphites
**FR_A**	14	contains sulphites
**FR_B**	13,5	*
**FR_C**	14	*
**SK_F**	9	natural residual sugar: 160 g/l; contains sulphites
**SK_G**	12	contains sulphites
**SK_H**	13	natural residual sugar: 77 g/l; contains sulphites
**SK_I**	13,5	rest. sugar: 1,7 g/l total acidity: 7,1 g/l contains sulphites
**SK_J**	14	contains sulphites
**SK_K**	12	contains sulfur dioxide

* - The sulphites content has not been provided by manufacture which means that it is <10 mg/L, otherwise the respective information should be provided by manufacturer (Regulation (EU) No 1169/2011 of the European Parliament and of the Council).
